# Healing from post-traumatic stress disorder through a synergy of science and spirituality

**DOI:** 10.4102/sajpsychiatry.v31i0.2560

**Published:** 2025-08-28

**Authors:** Name withheld by request

I am writing to share my journey through post-traumatic stress disorder (PTSD).

My experiences led me to blend science, medicine, and spirituality as complementary paths to healing. Having lived with depression and struggled with PTSD for 5 years, I have come to appreciate that medicine and science can offer powerful relief and structure, while spirituality brings meaning and depth to the healing process.

For years, depression felt like a relentless weight that blurred my sense of self and drained my energy. Initially, I approached treatment and healing through the clinical framework of psychiatry and psychology, trusting that science could provide answers. My first step was medication, starting with selective serotonin reuptake inhibitors (SSRIs) (Prozac, specifically), which helped correct some of my brain’s chemical imbalances related to serotonin and other neurotransmitters. Antidepressants became a vital support, as they steadied me enough to engage more deeply in therapy. But they were not a cure. Science taught me that depression often comes from a combination of biological, psychological, and environmental factors, and this understanding helped me realise that getting better was not simply a matter of willpower or mindset. Learning this was itself a kind of relief.

Beyond medication, cognitive-behavioural therapy (CBT) was another essential tool. Through CBT, I learned how to identify and reframe the thought patterns that often spiralled into negativity and despair. The CBT gave me tangible techniques to change my mental responses and offered a structured way to manage the seemingly endless cycle of depressive thoughts. It allowed me to shift my focus from just surviving to starting to gain insight into my inner world. Scientific interventions were creating the foundation for my healing, equipping me with ways to cope with the depressive state I was in.

But even as I stabilised, I felt a persistent emptiness that scientific therapies alone could not address. I recognised that therapy and medication were crucial to my treatment, yet I sensed something deeper that remained untouched – a sense of longing for meaning, peace, and connection that neither CBT nor medication could fully provide. This is when I began to explore spirituality and spiritual practices. I started with mindfulness meditation, which, although introduced to me as a therapeutic technique, gradually became a spiritual practice. As I sat with my breath, I began connecting with parts of myself that had been silenced or dismissed my creativity, innovation, and my being social. Mindfulness became more than just a clinical tool; it became a way to cultivate awareness and compassion for my own pain, and to reconnect with a sense of purpose beyond immediate relief.

One of the most profound aspects of spirituality for me was the practice of self-compassion and forgiveness. Living with PTSD often meant confronting not just fear and anxiety but also deep shame and self-criticism. Therapy helped me recognise these emotions; spiritual practices taught me how to respond with gentleness. Through compassion meditation and prayer, I began to soften towards my pain rather than fighting against it, which allowed me to release layers of guilt and hurt. This practice became a healing balm. Science had set the stage for this, but could not completely provide it on its own.

I see now that science and spirituality need not exist in opposition. Medicine and CBT created a stable structure for my mind and emotions, enabling me to engage in life again. Medication regulated my brain’s neurochemistry. Therapy empowered me with strategies to navigate the mental landscape of PTSD and depression. Spirituality, in turn, infused my recovery with purpose and meaning. It allowed me to see myself as more than just a person struggling with symptoms; I became someone on a journey of self-discovery and growth.

In my experience, medicine and spirituality work best not in isolation but as partners on the path to healing. Science and medicine gave me the tools to regain control of my life, while spirituality allowed me to embrace it fully. Together, they have brought me to a place of deeper healing – one that goes beyond simply treating symptoms to living intentionally with a sense of hope, wholeness, and peace.

Thank you for allowing me to share this with you. My hope is that by acknowledging the synergy between science and spirituality, we can open the door for more holistic approaches in mental health treatment, helping others find a full spectrum of support on their journeys.

